# Complete Osseointegration of a Retrieved 3-D Printed Porous Titanium Cervical Cage

**DOI:** 10.3389/fsurg.2020.526020

**Published:** 2020-11-26

**Authors:** Wimar van den Brink, Nancy Lamerigts

**Affiliations:** ^1^Department Neurosurgery, Isala, Zwolle, Netherlands; ^2^Emerging Implant Technolgies (EIT), Wurmlingen, Germany

**Keywords:** osseointegration, 3d printed titanium implant, porous titanium alloy, cervical spine fusion, bone ingrowth spinal cage

## Abstract

**Introduction:** Porous 3D-printed titanium has only recently been introduced for spinal applications. Evidence around its use is currently limited to animal studies and only few human case series. This study describes the histological findings of a retrieved EIT cervical cage, explanted 2 years after insertion.

**Materials and Methods:** The patient underwent a double level C4/C5 & C5/C6 anterior cervical decompression using EIT cervical cages without an anterior plate. Two years later the C6/7 level degenerated and began to cause myelopathic symptoms. In order to address the kyphotic imbalance of the cervical spine and fix the C6/7 level, the surgeon decided to remove the C5/6 cervical cage and bridge the fusion from C4 to C7 inclusive. The retrieved cage was histologically evaluated for bone ingrowth and signs of inflammation.

**Results:** MRI demonstrated spinal canal stenosis at C6/C7. Plain radiographs confirmed well-integrated cervical cages at 2 years postoperative. The peroperative surgical need to use a chisel to remove the implant at C5/C6 reconfirmed the solid fusion of the segment. Macroscopically white tissue, indicative of bone, was present at both superior and inferior surfaces of the explanted specimen. Histological evaluation revealed complete osseointegration of the 5 mm high EIT Cellular Titanium^®^ cervical cage, displaying mature lamellar bone in combination with bone marrow throughout the cage. Furthermore, a pattern of trabecular bone apposition (without fibrous tissue interface) and physiological remodeling activity was observed directly on the cellular titanium scaffold.

**Conclusion:** This histological retrieval study of a radiologically fused cervical EIT cage clearly demonstrates complete osseointegration within a 2-year time frame. The scaffold exhibits a bone in growth pattern and maturation of bone tissue similar of what has been demonstrated in animal studies evaluating similar porous titanium implants. The complete osseointegration throughout the cage indicates physiological loading conditions even in the central part of the cage. This pattern suggests the absence, or at least the minimization, of stress-shielding in this type of porous titanium cage.

## Introduction

Anterior cervical discectomy and fusion (ACDF) is a common procedure in cervical spine surgery. Various types of cage materials and designs, either combined with bone graft material and/or osteoinductive substances, are clinically in use. The size and shape of cages differ, depending on the design philosophy and technical production limitations of the material.

There are five critical areas in the clinical application of cages for spinal fusion that influence clinical results and imaging assessment capabilities. First, the occurrence of pseudoarthrosis (non-union), secondly subsidence and migration, thirdly suboptimal spinal balance, fourthly immunological reactivity due to the cage material and lastly the imaging distortion on MRI and CT scans. With the availability of 3-D printing of titanium in a cellular structure, it became possible to significantly address these clinical issues, being able to manufacture a structure that closely mimics bone, and that provides an optimal rough and porous scaffold for ingrowth of bone. EIT Cellular Titanium^®^ has been developed based on the results of various *in-vitro* and *in-vivo* studies, combining the various findings related to adequate pore size, shape, and porosity that would permit maximal bony ingrowth (1–9). The 80% porosity of cellular titanium warrants an elasticity modulus close to the bony environment. Furthermore, distortion on MRI and CT scans is minimized, especially when compared to the distortion observed with massive titanium, or trabecular metal implants. This allows for both a more detailed evaluation of the fusion process, and for evaluation of decompression of the neural structures (data on file VAL 2017-007). Because the 3D printed porous titanium material has only recently been introduced for spinal application, limited clinical studies and proof of fusion are available. In this retrieval study we describe the histological bone in growth pattern in a retrieved EIT Cellular Titanium^®^ cervical cage 2 years after implantation.

## Materials and Methods

The cervical implants applied in the patient were EIT cervical cages (EIT-spine GmbH, Wurmlingen), 3D printed porous titanium cages made from Ti6Al4V ELI powder. Selective Laser Melting (SLM) is used to produce the implants (3D Systems, Denver). The EIT implants consist of a porous titanium scaffold (pore size 700 μm, diamond shaped grid, porosity 80%) and small solid rims. The porous scaffold has a 0.25 mm off-set related to the solid rims, to ensure a direct implant scaffold-endplate contact. The cervical implant is anatomical shaped, with a tapered outline, uncovertebral sparings and a cranial anatomical dome to fit snugly the cervical intervertebral disc space (**Figure 3A**).

The MARS-MRI (Metal Artifact Reduction Sequence) enables a good evaluation of the cervical spine and neural structures with a minimum of artifact, which is also illustrated in the MRI of this patient, having two EIT cages at the levels C4/C5 and C5/C6 (**Figure 2**).

The EIT Cellular Titanium^®^ cervical cage at the level C5/C6 was extracted during revision surgery and was immediately stored in formaldehyde 4% buffer solution (**Figure 3B**).

The specimen was imbedded in resin (Technovit 9100), trimmed to reach the middle zone of the specimen (red line **Figure 3B**) and 4 sections from one half of the implant were made using a diamond band saw (Exakt). The thin-sections were grounded to a thickness of 25–35 μm stepwise with the Micro grinder machine (Exakt 400 CS, Norderstedt, Germany) and with grinding paper of different grain sizes. As last step the thin sections were polished with polishing paper (4,000 grain size).

The sections were stained with H&E (Gill2) and Masson's Goldner Trichrome. Before staining of the thin-sections the plastic resin was removed via deplast-solution. Therefore, the thin sections were incubated 2x in Xylene and further incubated 2x in Methoxyethyl acetate (MEA), followed by washing in acetone and rinsed with water. The deplasticized sections were stained, dehydrated and cover slipped with mounting medium. The stained thin-sections were scanned with the Zeiss Axio Scan Z.1 System with a 20x magnification.

## Case Report and Histological Results

**Figure 1:**
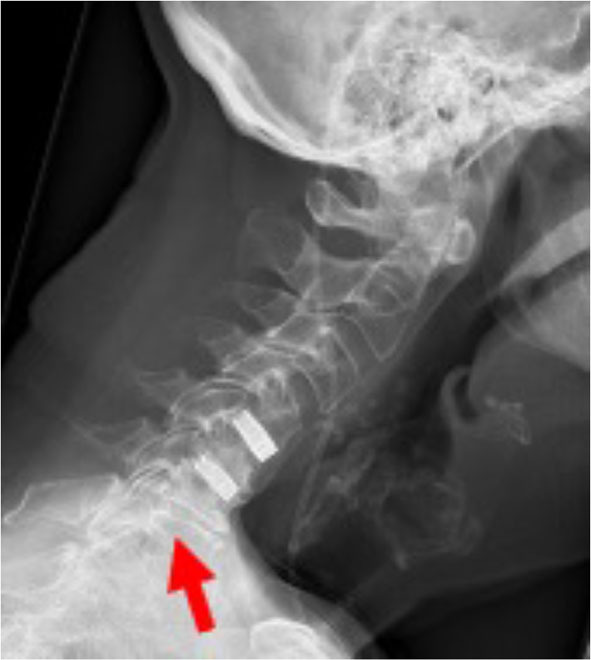
X-ray double level EIT CIF cage C4C5 and C5C6 2 years postop. Arrow indicates symptomatic level.

**Figure 2:**
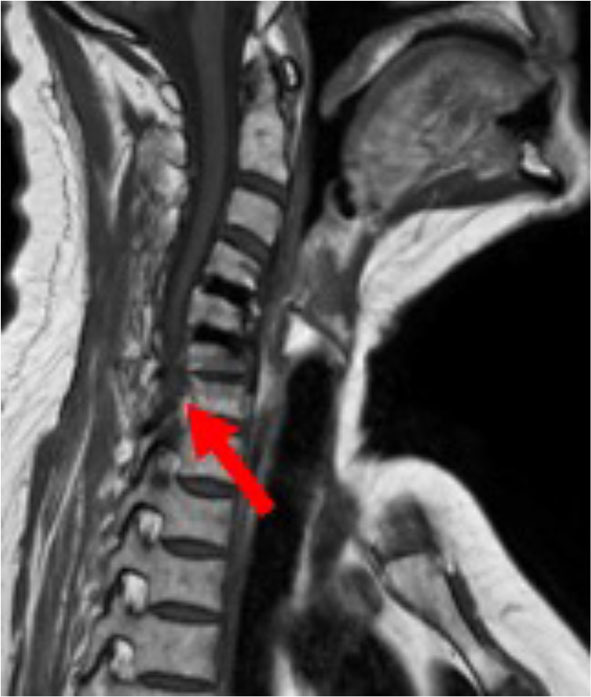
MRI double level EIT CIF cage 2 years postop Arrow indicates spinal stenosis C6/C7.

The patient (male, 70 yrs old) had a double level (C4/C5, C5/C6) ACDF using EIT Cellular Titanium^®^ cervical cages and no plate for 2 years before renewed symptomatology of the level below occurred (Figures 1, 2). Because of the cervical kyphotic malalignment, the surgeon (WBR) elected to sacrifice the C5/6 fused cervical level in order to treat the local symptomatology and restore the cervical lordosis over C5/C6-C6/C7.

The surgeon had to use chisels and force to extract the well-integrated cage.

**Figure 3:**
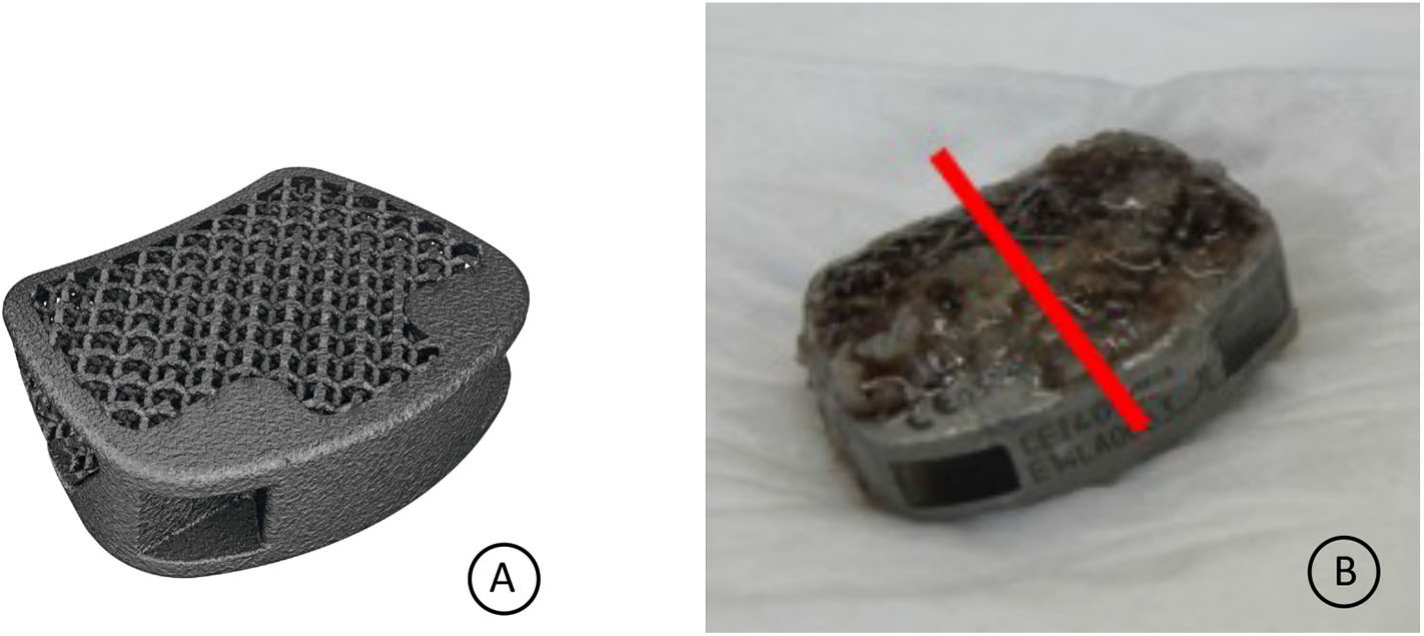
**(A)** EIT cervical cage. **(B)** Retrieved cervical cage specimen. The red line indicates the intersection line from which the histological sections were cut.

Macroscopic inspection of the retrieved cage already revealed white tissue, similar to bone, on both caudal and cranial cage-endplate contact areas (Figure 3B).

**Figure 4:**
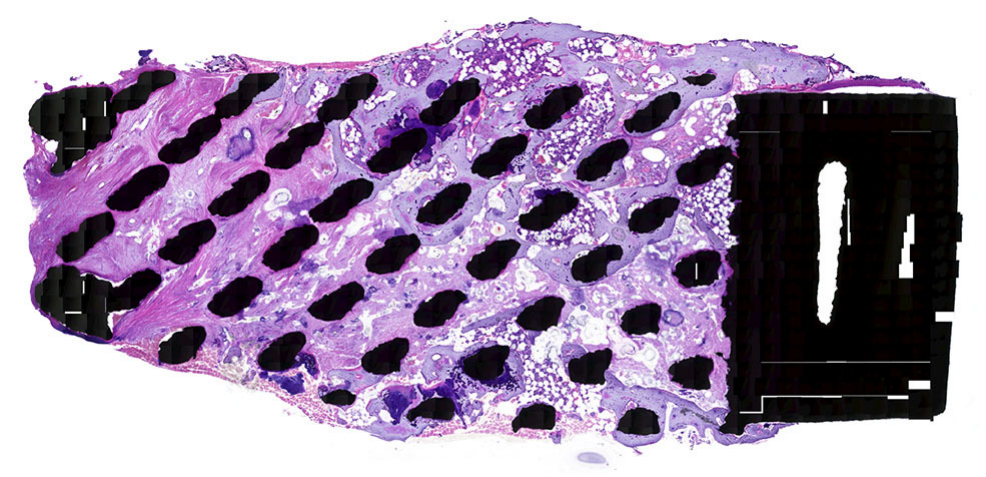
HE stained specimen, 20x magnification: This overview shows direct mature bone apposition onto the titanium struts (black spots), also present in the middle of the cage, indicating that the mechanical stimulation is transferred through the total cage.

In the HE stained specimen lamellar bone was found in close contact with the titanium surface and the bone extended throughout the cage from endplate to endplate (Figure 4). The microscopic analysis confirmed the infiltration of bony tissue in the anterior 2/3th of the cage, bridging the entire height of the 5 mm cage.

**Figure 5:**
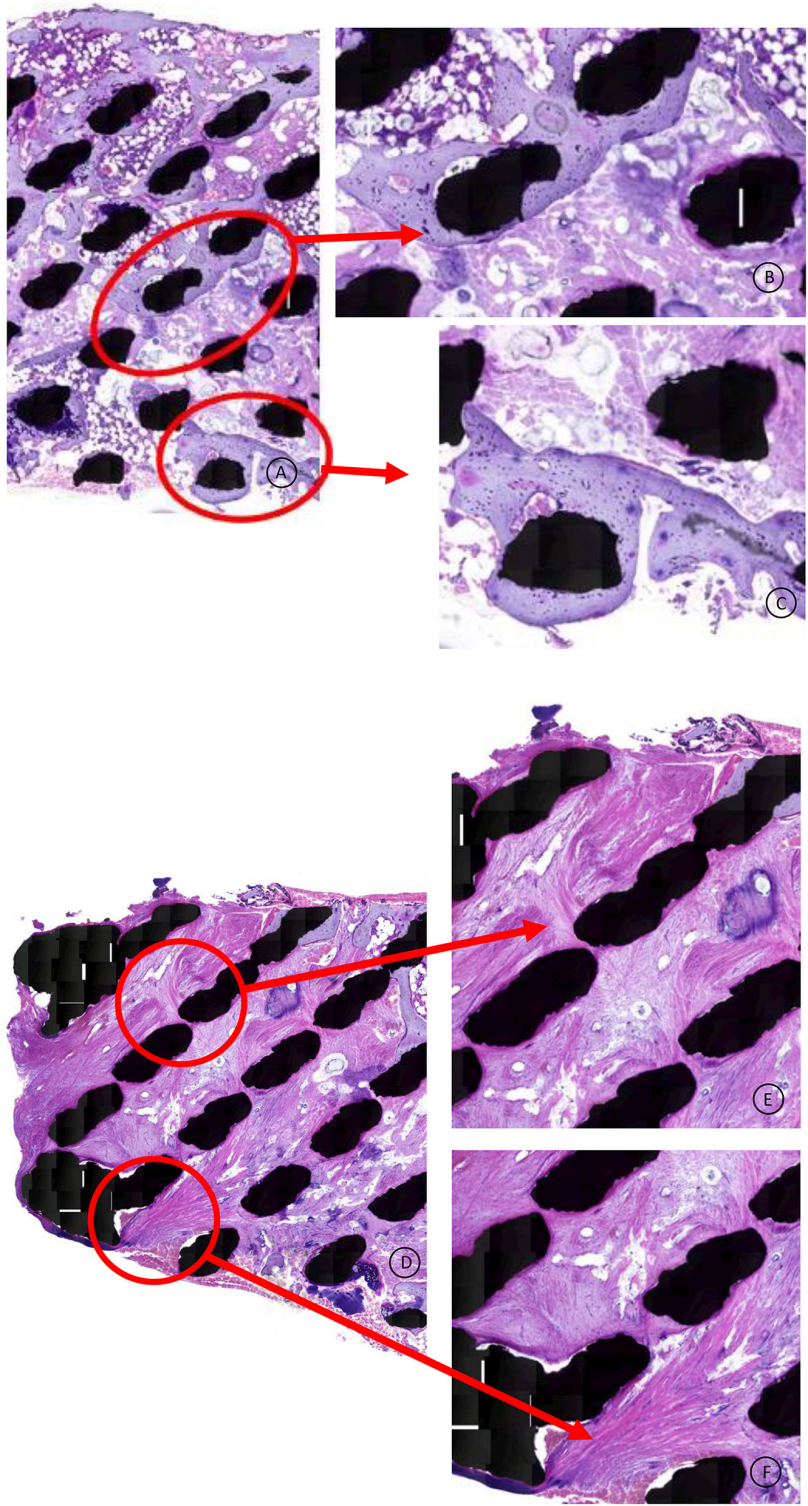
**(A–C)** HE stained specimen: Higher magnification from the cage middle and surface area. The lamellar bone has a mature, vivid appearance without any fibrous tissue intervening between the titanium and the bone. Healthy bone marrow can be observed throughout the scaffold. **(D–F)** HE stained specimen: Higher magnification from the posterior 1/3th of the cage. Dense collagen fibers are directed in a cranial to caudal trajectory, attaching to the titanium struts.

No fibrous tissue interface was found between the newly formed bone and the titanium struts (Figures 5A–C). The posterior part of the cage was infiltrated with dense fibrous tissue, demonstrating caudal-cranial directed collagen fibers (Figures 5D–F). Areas of bone marrow could be seen throughout the section, indicative of mature trabecular bone development.

**Figure 6:**
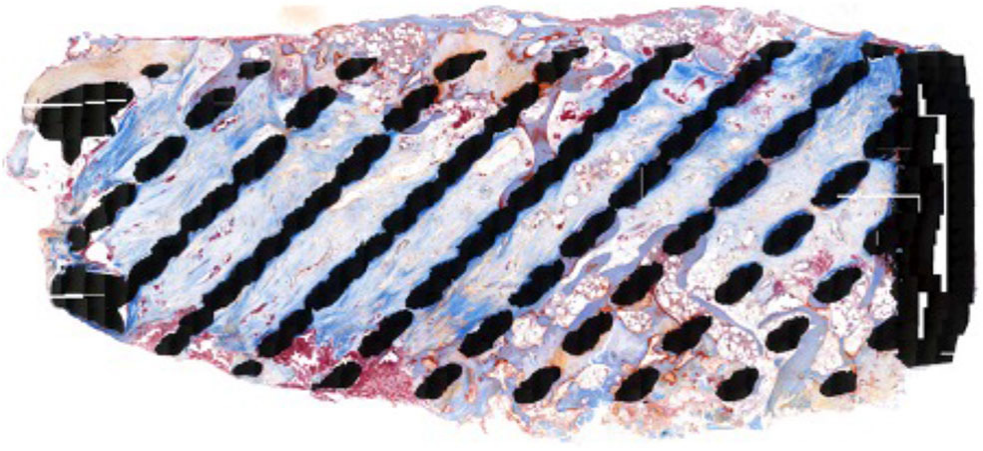
Masson Goldner Trichrome stained specimen, 20x magnification: In this staining the lamellar bone is colored light blue and young woven bone and osteoid has a red appearance.

**Figure 7:**
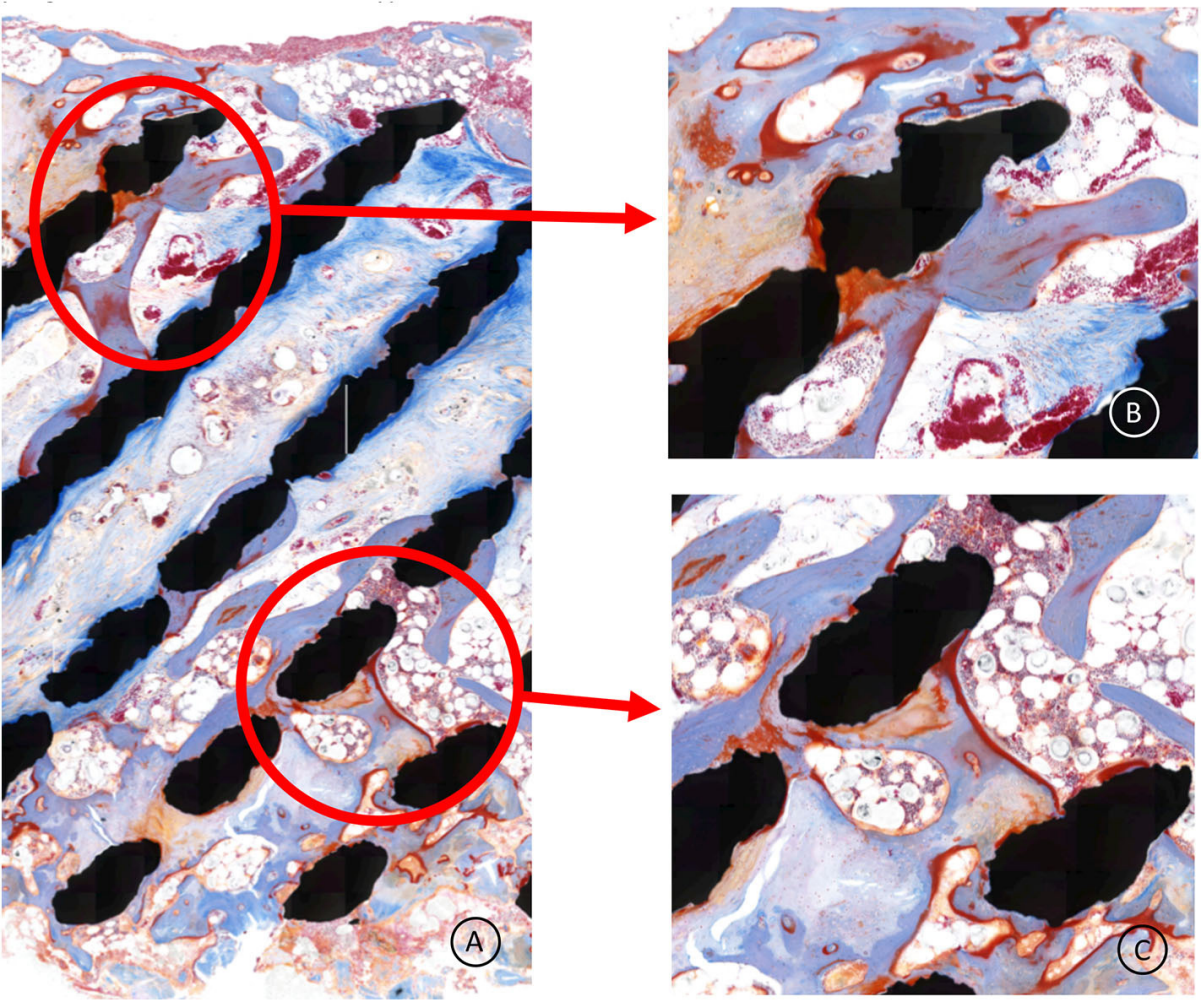
**(A–C)** Higher magnification of the cage central part; the bone (light blue) is remodeling, demonstrating adaptive reactivity. The red lining is osteoid, indicating active bone apposition by osteoblasts. No signs of inflammation are present. The whitisch bullous tissue is healthy bone marrow.

The Masson's Goldner Trichrome staining showed active bone remodeling in various areas throughout the section with patterns of adaptive reactivity and no appearance of signs of overloading (Figures 6, 7A–C). No inflammatory cells or tissue reactions could be observed.

## Discussion

Titanium alloy has a long track-record in orthopedic surgery for being a great biocompatible material, probably because of the ability of titanium to form a non-reactive TiO_2_ surface layer to resist corrosion and create a high surface energy that facilitates bone growth in and around the implant (10, 11). In the early years of spinal fusion surgery the applied solid titanium spinal cages tended to cause stress-shielding, subsidence and imaging issues. The development of PEEK spacers was a logical consequence (12), having more favorable mechanical characteristics with an elasticity modulus close to trabecular bone, avoiding the high rate of subsidence present in the solid titanium cages. Moreover, PEEK did not cause annoying artifacts neither on CT, nor MRI scans, allowing reliable postoperative evaluation of the performed surgery related to the extent of decompression, as well as the bony bridging through and around the cages in due time. Despite these characteristics of PEEK implants, the surface does not allow for bony adherence and thus pseudoarthrosis remains an ongoing issue (4, 8, 13).

EIT Cellular Titanium^®^, a 3D printed porous titanium scaffold, for application in cervical and lumbar cages, has been designed to tackle the most prominent critical clinical issues related to various cage materials, being the occurrence of pseudoarthrosis (non-union), subsidence, migration and imaging distortion.

The animal study of Wu et al., quantified the difference in direct bone contact between a porous titanium and PEEK cage in the cervical spine in a goat model. Whereas, the porous titanium cages had completely fused between the two vertebrae within 6 months' time, the PEEK cages lacked direct bone contact and exhibited abundant fibrous tissue formation (8). The configuration of the experimental porous titanium cage applied in the Wu study is very similar to the EIT scaffold in relation to pore size, diamond shape lattice and porosity.

Recently the prospective controlled clinical trial on single level ACDF without a plate conducted by Arts et al. (14) was published. This study confirmed the faster consolidation of EIT Cellular Titanium^®^ cervical cages in comparison to PEEK cages combined with autograft. Perhaps the difference of bony ingrowth throughout the porous cage instead of just surrounding the cage (such as PEEK and solid titanium implants) accounts for this difference according to the authors. Interestingly to note is despite the lack of bone graft or biomaterial in the porous titanium cages, the fusion process was accelerated in the early postoperative phase.

Titanium appears to have better bone integrative qualities in a specific porous configuration in comparison to a solid design. An ideal pore-shape in a “diamond-configuration,” a pore-size around 700 μm and a porosity of about 80% demonstrated the highest amount of osteogenic activity and osseointegration *in-vitro* and *in-vivo* (1, 2, 6, 7, 15). The porosity appears to have a positive effect on the differentiation markers, the number of osteocytes and the amount of maturated bone tissue in direct contact with the titanium surface (9, 16). The histology of this cervical retrieval cage is in line with the histological findings of the animal studies described above, being abundant bone apposition of lamellar, mature bone in direct contact with the trabecular struts of the porous scaffold.

Wolff's law states that bone will adapt to the loads under which it is placed (17) and cancellous bone aligns itself with internal stress lines. Mechanical loading is of key importance for osteocyte survival (18). The animal study of Lamerigts et al., assessed *in-vivo* the effect of various loading conditions on bone graft incorporation. The histology of the incorporation and remodeling process of morselized bone graft was quantified for various loading regimes. Non-loaded conditions resulted in disappearance of the graft material, leaving the critical size defect in the femoral condyle empty (19). The finding of healthy lamellar bone, in direct contact with the titanium struts throughout the retrieved cage strongly suggests that mechanical loading is also taking place in the center of the 5-mm high porous titanium cage.

Observing bone ingrowth more prominently in the anterior 2/3th of the cage, is in line with computed tomographic osteoabsorptiometry observations, indicating the largest force transmission in the peripheral marginal zones, with higher bone density in the anterior part of the vertebrae in the lower cervical levels (20).

The elastic behavior of a material is related to its elasticity modulus as well as its structural composition. A solid titanium cube will exhibit a different elastic behavior compared to a titanium lattice with 80% porosity. The “bulk elasticity modulus” of EIT Cellular Titanium^®^ is rather similar to PEEK (21). There was no sign of stress-shielding in the retrieval cage; stress-shielding is a significant negative side effect that can impair graft incorporation and fusion in cages with a box shaped design made of stiff material (22).

Magnetic Resonance Imaging is the diagnostic tool of choice when short or long-term postoperative complications occur after spinal surgery. Paramagnetic metal implants like titanium can provoke artifacts that impair the evaluation of MRI images and subsequent diagnostic and surgical work-up. Attributing significant porosity to a metal implant can reduce the MRI artifacts (23), which was also demonstrated in this case report with double level porous titanium cages.

## Conclusion

This histological retrieval study of a radiologically fused cervical EIT cage clearly demonstrates the complete osseointegration of the EIT Cellular Titanium^®^ scaffold 2 years postoperative. The scaffold exhibits a bone in growth pattern and maturation of bone tissue similar of what has been demonstrated in animal studies with comparable porous titanium implants. The complete osseointegration throughout the cage indicates physiological loading conditions even in the central part of the cage, suggestive of avoiding the occurrence of stress-shielding.

Results of ongoing clinical and pre-clinical research on the EIT Cellular Titanium^®^ Cages will further substantiate in the very near future the value of this new material in obtaining spinal fusion.

## Data Availability Statement

The datasets generated for this study are available on request to the corresponding author.

## Ethics Statement

Ethical review and approval was not required for the study on human participants in accordance with the local legislation and institutional requirements. Written informed consent for participation was not required for this study in accordance with the national legislation and the institutional requirements. Written informed consent was not obtained from the individual(s) for the publication of any potentially identifiable images or data included in this article.

## Author Contributions

WB delivered clinical material, including imaging and reviewed, and corrected the draft manuscript. NL organized histological preparation and evaluation of the specimen and prepared draft manuscript. All authors contributed to the article and approved the submitted version.

## Conflict of Interest

The authors declare that the research was conducted in the absence of any commercial or financial relationships that could be construed as a potential conflict of interest.
